# Leveraging mathematical models to predict and control T-cell activation

**DOI:** 10.1371/journal.pcbi.1013769

**Published:** 2026-04-27

**Authors:** Xabier Rey Barreiro, Jose Faro, Alejandro F. Villaverde

**Affiliations:** 1 CITMAga, Santiago de Compostela, Galicia, Spain; 2 Universidade de Vigo, Department of Systems Engineering and Control, Vigo, Galicia, Spain; 3 Universidade de Vigo, Department of Biochemistry, Genetics and Immunology, Vigo, Galicia, Spain; 4 Inflammatory and Infectious Diseases and Immune Disorders Research Group, Galicia Sur Health Research Institute (IIS Galicia Sur), SERGAS-UVIGO, Vigo, Galicia, Spain; Florida Atlantic University, UNITED STATES OF AMERICA

## Abstract

T-cell receptor (TCR)-mediated T-cell activation is a key process in adaptive immune responses. The complexity of this process has led to the development of different mathematical models that seek to describe and predict the conditions of antigen-TCR interactions required for TCR triggering and T-cell activation. These models are characterized by describing different sets of sequential molecular interactions and their kinetics, positing the generation of a final product as a necessary and sufficient condition for T-cell activation. Such modeling could provide an effective tool for simulating antigen recognition by T cells and, consequently, aid in the design of effective therapeutic strategies. However, it is necessary to previously assess the predictive capabilities of the proposed models when fitted to experimental data. As a first step towards this goal, in this work we examine the parameter identifiability and sensitivity of the published models of TCR-based T-cell activation. For each model, we consider different, often experimentally measured, output quantities and show how their availability affects the results. These analyses allow us to determine the ability of each model to correctly describe different experimental situations, and to establish to what extent these models can be applied to reliably predict and control T-cell activation by specific therapeutic targets.

## Author summary

The adaptive immune system is a highly specialized defense mechanism present in vertebrates. It produces a response that results from a complex set of interrelated cellular and molecular mechanisms. A key process is antigen-dependent activation of T lymphocytes, for which several mechanisms have been postulated. The corresponding mathematical models allow us to explore competing hypotheses, make quantitative predictions, and eventually aid in the design of strategies to control the immune response. To achieve these goals, it is essential that the models be identifiable and observable, that is, it must be possible to infer their parameters and state from output measurements. Furthermore, their response should exhibit an adequate level of sensitivity to certain key parameters. In this work, we evaluate the degree to which currently existing models possess these qualities. Our findings provide minimum requirements for the design of system identification experiments, allowing us to discard those models that cannot be successfully calibrated with a given experimental setup.

## Introduction

Adaptive immune responses to protein antigens (Ag) are based on interactions of T lymphocytes with target or Ag-presenting cells (APC) that lead to the activation of Ag-specific T cells. This lymphocyte activation is initiated by the binding of T-cell receptors (TCR) to their ligands, Ag-derived short peptides complexed with major histocompatibility complex proteins (pMHC) expressed on the membrane of APCs [1]. TCRs are membrane proteins composed of two chains, TCRα and TCRβ, each with two extracellular globular domains. They bind pMHC ligands by their two membrane-distal or variable domains but lack the capacity to directly transduce a signal intracellularly, owing to their very short intracellular tails. However, they can signal indirectly through their tight, non-covalent association with a membrane invariant multimer termed CD3, composed of two heterodimers (CD3ϵδ, and CD3ϵγ) and one homodimer (CD3ζζ) [1]. The four chains ϵ, δ, γ, and ζ, all have a single globular extracellular domain and a large intracytoplasmatic tail, with ζ having the largest one. The CD3 chains ϵ, δ, and γ have in their tail one signaling motif, containing two tyrosines, termed immunotyrosine activation motif (ITAM), while CD3 ζ chains contain two ITAMs [1,2]. There is evidence that TCR-pMHC binding induces a conformational change in the membrane proximal constant domain of the TCRβ chain that is transmitted to the CD3 extracellular domains, which drives the conversion of CD3 intracellular tails from an inactive to an active form [1,3–6]. This allows the phosphorylation of CD3 ITAMs, which, in turn, triggers an intracellular molecular signaling cascade that eventually leads to T-cell activation. The degree of that signaling depends to a variable extent on the pMHC concentration and the kinetic rate constants of both the TCR-pMHC interaction and the CD3 phosphorylation events [7–9]. Phosphorylated TCRs that reach the signaling state are denoted activated or triggered TCRs. Although these early signaling events constitute the focus of all phenotypic models analyzed here, full T-cell activation *in vivo* is also modulated by additional co-stimulatory and co-inhibitory pathways, such as those mediated by CD28 and CTLA-4. These mechanisms are not explicitly considered in the present study, which is restricted to published phenotypic models focused on early TCR signalling.

Due to the complexity of those processes, the relationship between these quantities and T-cell activation is not well understood yet. Over the years, numerous quantitative experimental data have been obtained (reviewed in [1]), inspiring the formulation of several mathematical models, most of them with a common core based on the kinetic proofreading concept [10]. In each of these models, an output can be defined that corresponds to the dynamics of a selected set of experimentally measurable quantities, known as *phenotypic* features of activated T cells [11], and that define a T-cell activation phenotype [11–13]. Here, ‘phenotype’ refers to the observable features of T-cell activation captured by those measurements (e.g., cytokine production or activation-marker expression) as a function of pMHC antigen dose (*L*_*T*_) and TCR–pMHC binding kinetics (discussed in more detail in [13]). However, it becomes challenging to determine which of those models (from now on, denoted phenotypic models) best describes T-cell activation.

Model parameters that cannot be directly measured are usually estimated by fitting the model output, that is, measurable quantities of the model dynamics, to experimental data [14,15]. However, finding the optimal fit does not guarantee that the corresponding parameter estimates are correct or meaningful. This issue is particularly important for those parameters that have a significant impact on the time-varying output, so that even a small variation in them has a big effect on model predictions. Therefore, before attempting to calibrate a model or use it to predict the behavior of a particular biological system under study, it should ideally be ensured that there are no model structural issues that can lead to unreliable and wrong model predictions [16,17]. Note that this does not imply that an unidentifiable model is always useless; it can still provide insights about the underlying biological mechanism, and furthermore, it may serve as the basis for obtaining an identifiable model.

As a step to achieve this ultimate goal, here we perform extensive analyses of most (if not all) published phenotypic models of T-cell activation. In particular, we examine the characteristics of these models with respect to their *parameter identifiability*, *state observability*, and *output sensitivity* to parameter values. Identifiability is the property that describes whether it is possible to determine the values of unknown parameters from the model output [18]. It is tightly linked with observability, which is a property that determines the feasibility of inferring the unmeasured dynamic variables, or states, from the model output [19], thus informing about a model’s ability to make reliable predictions about the state of a system over time. Here, we approach identifiability and observability from a *structural* viewpoint, i.e., we focus on obstructions to these properties that may arise from the differential and output equations of each model, rather than on limitations imposed by specific datasets, which are addressed by the *practical* counterparts of these properties [18]. Lastly, sensitivity analysis evaluates the extent the model output is affected by variations in parameter values [20–22]. These properties depend on which variables can be experimentally measured, also known as model outputs. Therefore, in our analyses, we consider as outputs different potentially measurable quantities like the amount of total, free, and activated or triggered TCRs per T cell (the latter as a surrogate of T-cell activation level). In addition, we also analyze as outputs two frequently measured quantities, the maximum ligand effect (*E*_*max*_) and the half-maximal effective ligand concentration (*EC*_50_).

In this article we address, thus, the following questions: First, which phenotypic models or model structures, i.e., which sets of dynamic and output equations can be used to predict T-cell activation reliably, considering the need to estimate their parameters from fitting procedures (identifiability)? Second, to what extent is the behavior of those phenotypic models influenced by the particular values of said kinetic parameters (sensitivity)? Finally, how can this combined knowledge about parameter identifiability and sensitivity be leveraged to determine which parameters are the best candidates to be tuned experimentally for driving T-cell activation in a controlled way?

### T-cell activation models

Mathematical models of T lymphocyte activation have been developed to elucidate how a signaling network translates Ag dose and kinetic parameters into a T-cell response [12,23,24]. These models aim to analyze the biochemistry of signaling in detail. However, given their complexity and the wide range of features they must account for, it has been suggested to reformulate these models into simplified versions known as *phenotypic models* [11]. Phenotypic models of T-cell activation focus on explaining the observable characteristics of the T-cell response in terms of a few key biochemical processes rather than the intricate biochemical details. The objective is to build a relatively simple yet efficient model capable of reproducing the observed phenotypes [13].

**Fig 1:**
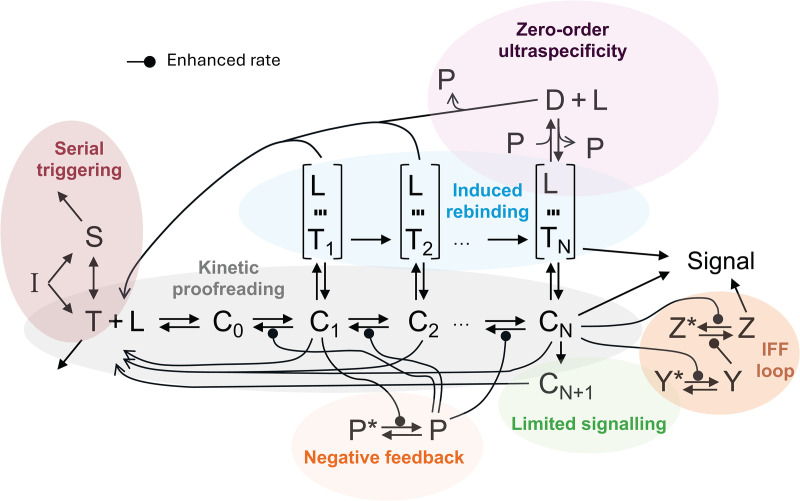
Master diagram of the models analyzed in this work. This figure is a schematic map showing the different sets of variables and interactions considered across the literature. Each model corresponds to a subset of the interactions/modules shown, and to facilitate their identification, motifs or mechanisms specific to the different published models are distinctly colored (see Fig A in S1 Text for the individual model schematics). *Common model variables*: *T* denotes free TCRs, *L* represents free pMHC ligands, *C* denotes TCR-ligand complexes, and *N* is an integer number that refers to the number of proofreading steps required for TCR triggering. *Zero-order Ultraspecificity*: *P* denotes free phosphatase and *D* denotes a complex of a phosphatase with a triggered TCR. *Negative Feedback*: *P* and P^*^ denote, respectively, active and inactive SHP-1 phosphatases. *Induced rebinding*: *T*_*i*_ denotes TCRs at proofreading step *i* that have recently unbound from their pMHC ligand but are still very close to it, in an intermediate state, such that they have the potential to rebind it again; this receptor-ligand state is indicated with three short lines. *Serial triggering*: *S* refers to TCRs in the membrane spare pool of T cells. *Limited signaling*: *C*_*N*+1_ refers to TCR-ligand complexes that underwent one proofreading step beyond the TCR-triggering one. *Incoherent feed-forward (IFF) loop*: *Y* denotes an intermediate metabolite required to enhance the transformation of a signaling molecule (denoted *Z*) from an inactive state to an active state.

Any model designed to describe T-cell activation quantitatively must be capable of considering and quantifying the following key features of the adaptive immune response: (1) sensitivity to detect foreign pMHCs at very low concentrations amid a significantly higher abundance of self-pMHC ligands, (2) discrimination or specificity to distinguish between foreign and self-ligands and not allow self-peptides to trigger an immune response, and (3) a short reaction time to foreign peptides, ensuring these requirements are met promptly [25]. This section briefly describes the T-cell activation phenotypic models we have found in the literature (listed in Table 1). A general diagram of the different mechanisms or regulatory motifs they describe is depicted in Fig 1, which presents a synoptic view of how the different regulatory motifs postulated by the distinct models relate to each other. For each model, its specific conceptual scheme and equations are given in Fig A in S1 Text.

**Table 1:** Phenotypic models analysed in this work.

Model	Short name	Reference
Occupancy	**Occ**	[26]
Kinetic proofreading (McKeithan)	**KPR-1**	[10]
KPR with limited signaling	**KPR-LS**	[11]
KPR with sustained signaling	**KPR-SS**	[11]
KPR with induced rebinding	**KPR-IR**	[27]
KPR with limited signaling and		
incoherent feedforward loop	**KPR-LS-IFF**	[12]
KPR with negative feedback I	**KPR-NF1**	[25]
KPR with negative feedback II	**KPR-NF2**	[2]
KPR with stabilizing activation chain	**KPR-SAC**	[23]
Zero-order ultrasensitivity	**ZU**	[28]
KPR with zero-order ultrasensitivity	**KPZU**	[28]
KPR with concentration compensation	**KPC**	[28]
Serial triggering	**ST**	[29]

### Occupancy Model (*Occ*)

The occupancy model [26] posits that T-cell activation is proportional to the concentration of TCR-pMHC complexes. It assumes, therefore, that TCRs reach a signaling-competent state immediately after binding a pMHC.

### Kinetic Proofreading Models (*KPR*)

The kinetic proofreading (KPR) mechanism was initially proposed by J. Hopfield [30] and J. Ninio [31] to explain how enzymes lead to correct products over incorrect ones with high accuracy. A couple of decades later, this mechanism was adapted by McKeithan to the case of T-cell activation as a possible way to explain how T cells achieve precise discrimination between cognate foreign and self-pMHC ligands, despite the overwhelming presence of the later [10]. This concept posits that the interaction between TCRs and pMHC ligands does not immediately result in signaling upon docking. Instead, the TCR must undergo a series of post-translational modifications and spatial rearrangements before initiating effective signaling into the cell. During this process, if the TCR-pMHC complex dissociates prematurely due to high unbinding rates, the signaling process is halted and restarted, thereby preventing incorrect activation. Only TCR-pMHC complexes with sufficiently small unbinding rates persist long enough to complete all the necessary steps and trigger an effective immune response. Over the years, different versions and extensions of McKeithan’s original KPR model have been proposed; they are described below.

### KPR McKeithan (*KPR-1*)

This model is perhaps the simplest representation of lymphocyte receptor activation through kinetic proofreading [10]. According to it, TCR binds reversibly to pMHC, and bound TCRs initiate a sequential process of phosphorylation at the CD3 intracellular ITAM motifs. At each TCR phosphorylation stage, if the pMHC unbinds the TCR, this reverts to its basal unmodified state. Only those pMHC-bound TCRs that have gone through a threshold number of steps (*i.e.*, that have accumulated a threshold number of phosphorylations) become triggered and start signaling T-cell activation.

### KPR with Limited Signaling (*KPR-LS*)

This model extends the *KPR* model by postulating that pMHC-bound TCRs that reach the triggering state can be further phosphorylated into a state in which they lose the signaling ability before unbinding pMHC. Its development was motivated by experimental observations on serial triggering suggesting that efficient T-cell activation requires that the TCR-pMHC dwell time lies within an optimal range [32]. This model, proposed in [11], incorporates into a *KPR* framework the serial triggering feature that triggered TCRs signal T cells for a fixed period of time, leading to an optimum T-cell activation level with respect to the TCR-pMHC dissociation time.

### KPR with Sustained Signaling (*KPR-SS*)

This model was developed following experimental observations indicating that at high ligand densities optimal T-cell activation also takes place at extended TCR-pMHC dwell times and not only within an optimal range [33]. Thus, in contrast to KPR-LS, where triggered pMHC–TCR complexes can undergo an extra phosphorylation step that renders them signaling-inactive, in the *KPR-SS* model signaling by triggered TCRs is sustained after unbinding: TCR-pMHC complexes that have reached the triggering state not only can reversibly dissociate, but recently dissociated triggered TCRs remain in a signaling state for some finite time before reverting to the basal state [11].

### KPR with Induced Rebinding (*KPR-IR*)

The *KPR-1* model predicts high specificity but reduced receptor sensitivity. This occurs because, as the number of phosphorylation steps needed to complete the phosphorylation of a bound TCR and trigger it (phosphorylation threshold) increases, so does the time required for TCR triggering, and therefore the proportion of TCRs that remain bound to pMHCs decreases exponentially. Thus, there is a trade-off such that a larger phosphorylation threshold leads to higher specificity but lower sensitivity, rendering T cells less capable of responding to low concentrations of specific antigens. In order to correct for this shortcoming, the *KPR-IR* extension accounts for the possibility that recently unbound TCRs remain for a time in a state and at a distance that facilitate their subsequent binding to the same pMHC [27]. This extension assumes, thus, that the initial binding of a pMHC ligand to a TCR induces in the receptor some changes, such as TCR clustering and/or conformational alterations, that could significantly increase rebinding to that pMHC. This mechanism enhances both sensitivity and specificity. We analysed an approximate version of this model derived in [24]. In this approximation, we assume that rebinding after unbinding occurs fast enough so that bound states and unbound but encounter-oriented states can be merged into one effective state [34].

### KPR with Limited Signaling and Incoherent Feed-Forward Loop (*KPR-LS-IFF*)

Shortly after proposing the *KPR-LS* model, its authors performed a series of *in vitro* experiments, systematically analyzing T-cell responses to 10^6^-fold variations in both pMHC concentration and TCR-pMHC 3-dimensional effective affinity. They observed four key phenotypic features related to dose-response and peak amplitude of the response [12]. This led them to explore systematically thousands of variants of the *KPR* model of increasing complexity to find those able to explain the observed features of T-cell responses. They concluded that a *KPR-LS* model with a coupled incoherent feed-forward (IFF) loop is the simplest model able to explain the four phenotypic features. The feed-forward loop consists of a signaling network where pMHC-bound, triggered TCRs activate two metabolic reactions: one that indirectly activates the final signaling process and another one that directly inhibits it (detailed in Fig 1).

### KPR with negative feedback, variants I and II (*KPR-NF1*, *KPR-NF2*)

Experimental findings showing that the Src homology 2 domain phosphatase-1 (SHP-1) has an important impact on T-cell activation by regulating the phosphorylation level of pMHC-bound TCRs [35] led to another extension of the basic *KPR* model. According to it, TCRs in complexes *C*_1_ of the proofreading chain activate a phosphatase which, in turn, reverses with similar rate constant each phosphorylation step in the proofreading chain [25]. This model, KPR with negative feedback I (in short, *KPR-NF1*), can explain the above mentioned three key properties in T-cell signaling: speed, specificity, and sensitivity. Recent experimental results prompted the same authors to modify the *KPR-NF1* model by postulating that the TCRs in the last *r* complexes of the proofreading chain can contribute to signaling the T cell, but with increasing contribution from *C*_*N*−*r*+1_ to *C*_*N*_ [2]. Notice that this new model, denoted here *KPR-NF2*, has the same ODEs as the *KPR-NF1* model.

### KPR with stabilizing/destabilizing activation chain (*KPR-SAC*)

This extension addresses the characteristic trade-off between T-cell specificity and sensitivity by introducing state-dependent kinetics along the proofreading chain. Specifically, for TCR-pMHC complexes bearing agonist peptides, the model postulates an increasing stabilization of the bound state, and facilitation of forward progression as the complex advances through the chain, that is: $k_{\;off}(i)>k_{\;off}(i+1)$ and *k*_*p*_(*i*) < *k*_*p*_(*i* + 1) (see Fig A(h) in S1 Text). For non-agonist peptides, the opposite trends are assumed, leading to a progressive destabilization and slowdown of proofreading: $k_{\;off}(i)<k_{\;off}(i+1)$ and *k*_*p*_(*i*) > *k*_*p*_(*i* + 1) along the proofreading chain [23].

### Zero-order ultraspecificiy

More than forty years ago, Goldbeter and Koshland [36] proposed the so-called zero-order ultrasensitivity (*ZU*) mechanism to explain enhanced sensitivity to changes in enzyme concentration in an enzymatic system in which the enzyme operates in saturating conditions with respect to substrate. Recently, the *ZU* mechanism was applied to T-cell activation by TCR-pMHC complexes [28]. Based on that mechanism, the authors presented three models of increasing complexity, from a basic *ZU* model to a model combining the *ZU* and the *KPR* mechanisms, all of which are briefly described below.

### Zero-order ultrasensitivity (*ZU*)

In this adaptation of the zero-order ultrasensitivity mechanism to TCR-driven T-cell activation, pMHC ligands play the same role as the first enzyme in the original model, and TCRs and phosphorylated TCRs act as substrate and product, respectively. Here, the role played by the second enzyme in the original model is ascribed to a phosphatase. Thus, when nearly all cognate pMHC ligands are bound to TCRs, the sensitivity of the system to small changes in ligand concentration is highly enhanced. Unlike multistep proofreading mechanisms, zero-order ultrasensitivity increases sensitivity and enables rapid responses, while compromising specificity, that is, reducing the ability to discriminate against self-antigens [28].

### KPR with zero-order ultraspecificity (*KPZU*)

The KPR with zero-order ultraspecificity model, *KPZU* for short (*GKP* in the original paper [28]), combines the kinetic proofreading with the zero-order ultrasensitivity mechanism. This combination enables it to balance specificity, sensitivity, and speed effectively.

### KPZU with concentration compensation (*KPC*)

The *KPC* model simplifies the *KPZU* model by assuming that the same molecule regulates both TCR phosphorylation and dephosphorylation [28]. This allows for ligand concentration compensation, enabling the system to remain insensitive to high concentrations of non-target ligands while maintaining sensitivity to low concentrations of target ligands.

### Serial triggering (*ST*)

In 1995, Valitutti and Lanzavecchia found that on average a single pMHC molecule can engage a large number of TCRs, triggering their phosphorylation and internalization (downregulation), and eventually activate T cells [37]. Based on this finding, they proposed the serial triggering (*ST*) conceptual model, according to which a small number of pMHC complexes can activate a T cell by binding, each of them, to a TCR long enough to trigger its phosphorylation and downregulation, and then dissociating from this receptor and binding to another TCR, thereby sequentially triggering many different receptors. Subsequently, Ohashi and *col* developed a mathematical model to analyze this finding [38]. They proposed the following dimerization model of TCR-pMHC complexes to explain the observed kinetics of TCR downregulation, and hence of serial triggering:


$$T+L\mathrm{\backslash xleftrightharpoons}[k_{off}]k_{on}C0$$ (1)

$$C_{0}+C_{0}\mathrm{\backslash xleftrightharpoons}[k_{r}]k_{f}C^{*}\overset{k_{a}}{\to }2T_{P}+2L$$ (2)

where *T* is the free TCR density on a T cell, *L* is the ligand (pMHC) density on an APC, and *C*_0_, C^*^ and *T*_*P*_ are the densities of TCR-pMHC complexes, *C*_0_ dimers, and triggered TCRs, respectively. Assuming that *C*_0_ formation reaches fast a quasi-steady state with *T* and *L*, and assuming also a quasi-steady state approximation for dimers, the authors arrived at the following equation for triggered TCRs [38]:


$$\frac{dR_{T}(t)}{dt}=k_{\;eff}T(t{)}^{h}L^{h},$$ (3)

where $k_{\;eff}$ is an effective binding rate defined by $k_{\;eff}=h\;k_{a}\frac{k_{f}}{k_{r}+k_{a}}{\left(k_{\;on}/k_{\;off}\right)}^{h}$, and *h* = 2.

To account for experimental results not explained by this model, other authors extended it a few years later by incorporating additional features of TCR dynamics [29]. In the enhanced *ST* model, TCRs are partitioned into two subsets, one consisting of the TCRs within the membrane interface or contact area between a T cell and an APC (named the interface pool), and another with those TCRs outside the interface area (named the spare pool). Free TCRs are assumed to diffuse freely from one pool to the other, and to turn over between the membrane and the cytoplasm. In this model, only TCRs of the interface pool can interact with pMHC molecules presented by an APC, and they do it irreversibly, with effective kinetic rate $k_{\;eff}$, as defined above, and kinetic order *h* > 1, leading to triggered (phosphorylated) TCRs, which are then internalized with rate *k*_*i*_ [29].

The original mathematical formulation of the *ST* model suffers, however, from some inconsistencies, which we discussed in [24]. To remove them, we reformulated the model by considering that the inter-pool diffusion of free TCRs takes place with rate ϕ proportional to the ratio of the interface to spare pool areas, and that receptors in each pool turnover between the membrane and the cytoplasm with rate σ, also proportional to the respective areas of the two TCR pools.

### Results

The structural identifiability and observability (SIO) of a model depend on the quantities that can be measured (output variables). The five most commonly measured quantities in T-cell activation research are *T*_*T*_ (the cell density of total TCRs); *T*(*t*) (the cell density of free TCRs); *R*(*t*) (*t*he total amoun*t* of triggered or activated TCRs per cell, except in the *KPR-LS-IFF* model where it is the cell density of the signaling molecule P); *E*_*max*_ (the maximal effective response); and *EC*_50_ (the ligand concentration leading to a half-maximal response). The latter two quantities can be used as outputs if we know the mathematical expression that relates them to parameters and state variables. Recently, we have derived those expressions [24], and here we make use of them in the identifiability and observability analyses of the models for several output configurations. We also analyzed in each model the sensitivity of the response to changes in key parameters.

#### Identifiability and observability

When output information in complex models is limited, we can expect that a number of unknown parameters will be unidentifiable [39]. Identifiability, however, can be improved by obtaining data for additional quantities, either state variables or parameters. To take this possibility into account, we also conducted identifiability analyses assuming that several measurements are available simultaneously. Specifically, we considered the possibility of measuring *R*(*t*), *T*(*t*), *T*_*T*_, *E*_*max*_, and *EC*_50_ either alone or in some combination. Likewise, we explored the possibility that the ligand-receptor binding rate ($k_{\;on}$) or the forward rate of proofreading (*k*_*p*_) are also known.

For each of the models, we determined which parameters and state variables are structurally globally identifiable (SGI), locally identifiable (SLI), and non-identifiable (SNI) for different output configurations. The results are summarized in Table 2. There the number of parameters and state variables that are SGI, SLI, and SNI are indicated. Detailed results are given in Tables A and B in S1 Text. Table A shows the identifiability results when the assumed measured outputs are *T*(*t*), *T*_*T*_ and *R*(*t*) (or a combination of *R*(*t*) with *T*(*t*) or *T*_*T*_) either alone or assuming *t*hat parame*t*ers $k_{\;on}$ and *k*_*p*_ are also known. Table B shows the results when the outputs are *E*_*max*_ or *EC*_50_, either alone or combined with *T*(*t*), *T*_*T*_, $k_{\;on}$ and *k*_*p*_. In what follows we describe the results for the different output configurations, focusing on the most relevant ones. Tables C and D in S1 Text rank, respectively, the models and output configurations that are more informa*t*ive in terms of identifiability.

**Table 2:** Number of parameters and initial conditions of each model that are structurally globally identifiable (SGI), locally identifiable (SLI), and non-identifiable (SNI) (indicated in each column as: #SGI/#SLI/#SNI) when the indicated specific variables are assumed to be measured; n.d., not done.

Models	Variables assumed to be measured (model output)								
	T_T_	R(t)	R(t) & T_T_	T(t)	R(t) & T(t)	E_max_	E_max_ & T(t)	EC_50_	EC_50_ & T(t)
**Occ**	-/-/4	1/-/3	3/-/-	1/-/3	3/-/-	-/-/5	4/-/-	-/5/-	4/-/-
**KPR-1**	-/-/6	-/-/7	-/6/-	1/-/5	6/-/-	-/-/7	-/1/5	-/-/7	1/2/3
**KPR-LS**	-/-/8	7/2/-	8/-/-	1/-/7	8/-/-	-/-/9	-/-/8	-/-/9	1/2/5
**KPR-SS**	-/-/8	-/9/-	8/-/-	-/8/-	8/-/-	-/-/9	-/8/-	-/9/-	-/8/-
**KPR-IR**	-/9/-	8/2/-	-/9/-	-/9/-	9/-/-	-/10/-	-/9/-	-/10/-	-/9/-
**KPR-LS-IFF**	-/-/16	-/5/11	-/12/3	1/-/15	-/12/3	-/4/13	-/4/12	-/-/17	-/-/16
**KPR-NF1**	-/-/12	-/10/3	-/9/3	1/-/11	-/9/3	-/-/13	-/1/11	-/-/13	-/3/9
**KPR-NF2**	-/-/13	-/11/3	-/10/3	1/-/12	-/10/3	-/-/14	-/2/11	-/-/14	-/3/10
**KPR-SAC**	-/-/8	-/9/-	-/8/-	-/8/-	8/-/-	-/-/9	-/8/-	-/-/9	-/8/-
**ZU**	-/-/10	-/10/-	-/9/-	-/10/-	9/-/-	-/11/-	-/10/-	n.d.	n.d.
**KPZU**	-/-/12	-/12/-	-/11/-	-/12/-	-/11/-	-/12/-	-/11/-	n.d.	n.d.
**KPC**	-/-/11	-/11/-	-/10/-	-/11/-	10/-/-	-/-/11	-/10/-	-/11/-	-/10/-
**ST**	-/6/2	-/6/2	-/5/2	-/4/4	-/5/2	-/-/9	-/5/3	-/-/9	-/6/2

#### (a) Identifiability when measuring the response *R*(*t*) (triggered TCRs).

If the response *R*(*t*) is the only output measured, seven of the models are fully identifiable (locally or globally), namely, *KPR-LS*, *KPR-SS*, *KPR-IR*, *KPR-SAC* and the three models of the *zero-ultraspecificity* family. Surprisingly, in contrast with the *KPR-LS* model, the *KPR-LS-IFF* variant has eleven unidentifiable parameters. Likewise, in the *KPR-NF1* and *KPR-NF2* models one parameter and the variables involved in the regulation of phosphatase SHP-1 activity are unidentifiable, and in the *Occ* and *ST* models some parameters and variables are also unidentifiable. Interestingly, in this case the simplest kinetic proofreading model (*KPR-1*) is not identifiable. However, when either *T*_*T*_, the total number of TCRs, or *T*(*t*), the total number of free TCRs, is measured in addi*t*ion to *R*(*t*), the *Occ* and *KPR-1* models become globally identifiable, and seven parameters in *t*he *KPR-LS-IFF* model previously unidentifiable become identifiable. Lastly, measuring $k_{\;on}$ and/or *k*_*p*_ in addition to *R*(*t*) has no positive impact on the identifiability, except in the *KPR-1* model, where all parameters become identifiable.

#### (b) Identifiability when measuring *T*_*T*_ (total number of receptors) or *T*(*t*) (instantaneous number of free receptors).

Measuring only *T*_*T*_ yields the worst results in terms of identifiability: all parameters in all models, except for the *KPR-IR* and *ST* models, are unidentifiable. Intuitively, it seems logical that *T*_*T*_ be the least informative measurement: being the sum of all TCRs, free and bound, its aggregate nature does not provide detailed information about individual variables, whose effects can be offset, thus making them indistinguishable.

However, while measuring only *T*_*T*_ is not enough for parameter identification, results for *Occ*, *KPR-1* and *KPR-LS-IFF* models considerably improve by combining it with *R*(*t*): many parameters that in *t*hose models were unidentifiable from *T*_*T*_ or *R*(*t*) alone become at least locally identifiable when both outputs are known (Table 2).

When *T*(*t*) is the measured output, all parameters of the *KPR-SS*, *KPR-IR*, *KPR-SAC*, *ZU*, *KPZU* and *KPC* models are identifiable, albeit only locally. In contrast, in the *Occ*, *KPR-1*, *KPR-LS KPR-LS-IFF*, *KPR-NF1* and *KPR-NF2* models, when only *T*(*t*) is measured $k_{\;on}$ is the only identifiable parameter (globally in this case), and identifiability is not improved by knowing additionally $k_{\;on}$ or *k*_*p*_; however, measuring *R*(*t*) in addition to *T*(*t*) makes all parameters identifiable in these models, except those parameters linked to the SHP-1 regula*t*or in the *KPR-NF1* and *KPR-NF2* models, and those linked *t*o *Y*, the activator of the signaling process, in the *KPR-LS-IFF* model. The ST model is singular in that measuring only *T*(*t*) yields four identifiable parameters (out of eight), while measuring also *R*(*t*) increases by one the number of identifiable parameters.

In general, knowing the values of $k_{\;on}$ and/or *k*_*p*_ in addition to the output variables indicated in Table A in S1 Text does not improve or improve a little the identifiability of other parameters, with the important exception of the *KPR-1* model (see above).

#### (c) Identifiability when measuring *EC*_50_ and *E*_*max*_.

Thus far, we have presented results for measurements that correspond directly to a single basic variable or a combination of two basic variables common to all models. The quantities *E*_*max*_ and *EC*_50_, however, are derived variables, defined by particular parameter-dependent expressions, specific to each model. For the *Occ*, *KPR-1* and *KPR-LS* models, these expressions can be found in the literature, as well as *E*_*max*_ for the *KPR-SS* model [11]. For the remaining models, recently, we have derived the corresponding expressions for *E*_*max*_ and *EC*_50_ (except *EC*_50_ in the *ZU* and *KPZU* models) [24] following the procedure outlined in Box 1. They are provided in S1 Text, section 2.

Importantly, measuring only *EC*_50_ or *E*_*max*_ gives poor identifiability results that are only a little better than measuring only *T*_*T*_ (Table 2). Thus, when measuring only *EC*_50_ all parameters are unidentifiable in seven out of eleven models (*KPR-1*, *KPR-LS*, *KPR-LS-IFF*, *KPR-NF1*, *KPR-NF2*, *KPR-SAC*, and *ST*), in contrast to four models where all parameters are locally identifiable (*Occ*, *KPR-SS*, *KPR-IR*, and *KPC*). However, when *T*(*t*) is measured in addition to *EC*_50_, parameters $k_{\;on}$ and $k_{\;off}$, and *L*_*T*_ become identifiable in the above unidentifiable models, except in *t*he *KPR-LS-IFF* model, where all parameters remain unidentifiable.

The case of *E*_*max*_ is even worse, resulting less informative than *EC*_50_ for all models. Thus, in this case only the *KPR-IR*, *ZU*, and *KPZU* models are fully locally identifiable, and in the *KPR-LS-IFF* model four out of seventeen parameters are locally identifiable. Adding measurements of *T*(*t*) considerably improves the results, so tha*t* not only the *Occ*, *KPR-SS*, *KPR-SAC* and *KPC* models become now fully identifiable, but in the *ST* model five parameters out of nine become locally identifiable, one parameter in the *KPR-1* and *KPR-NF1* models, and two parameters in the *KPR-NF2* model. All parameters in the *KPR-LS* model remain unidentifiable.

### Sensitivity analysis

To perform sensitivity analysis, we fixed a set of reference parameter values taken from the literature (Table 3) and focused on a subset of parameters usually considered more relevant for T-cell activation, namely, $k_{\;off}$, $k_{\;on}$, *k*_*p*_ and *L*_*T*_, common to all models—except the *ST* model that does not include $k_{\;off}$ and *k*_*p*_, and the *Occ* model that does not include *k*_*p*_. We varied the values of these parameters within biologically reasonable ranges spanning several orders of magnitude (Table E in S1 Text), and quantified the corresponding sensitivity of *R*(*t*). Also, given that the *KPR-NF1* and *KPR-NF2* models include a dephosphorylation mechanism —contributed by a basal rate constant *b* and a variable rate γP(t)—, in these two models the sensitivity of *R*(*t*) was also analyzed with respect to *b* and γ. Moreover, since the dynamics of *P*(*t*) (active phosphatase) is key for both models’ behavior, we also analyzed the sensitivity of *P*(*t*) to parameters $k_{\;off}$, *b* and γ. Naturally, other analyses could be performed as well; however, while it would be possible to compute the sensitivities of the outputs of all models to all parameters, many of the results would be of limited interes*t*.

**Table 3:** Parameter values used in the sensitivity analyses.

Name	Value	Units	Description and references
$k_{\;on}$	5.0 × 10^−5^	*s*^−1^ (*molecules/cell*)^−1^	TCR-pMHC binding rate [23]
$k_{\;off}$	1.0 × 10^−2^	*s* ^−1^	TCR-pMHC unbinding rate [23]
*T*_*T*_	2.0 × 10^4^	*molecules/cell*	Total number of TCRs per T cell [23]
*L*_*T*_	1.0 × 10^2^	*molecules/cell*	Total number of pMHC ligands per APC [23]
*k*_*p*_	1.0 × 10^0^	*s* ^−1^	Phosphorylation rate in KPR models [23]
*P*_*T*_	6.0 × 10^5^	*molecules/cell*	Total number of SHP-1 molecules per T cell [2,35]
α	2.0 × 10^−4^	*s* ^−1^	SHP-1 activation rate [23]
β	1.0 × 10^0^	*s* ^−1^	SHP-1 deactivation rate [23]
β/α	5.0 × 10^2^		SHP-1 deactivation-activation ratio for KPR-NF2 [2]
*b*	4.0 × 10^−2^	*s* ^−1^	Spontaneous dephosphorylation rate [25]
γ	4.4 × 10^−4^	*s* ^−1^	Dephosphorylation rate by SHP-1 [2]
ϕ	9.0 × 10^−2^	*s* ^−1^	Phosphorylation rate of *C*_*N*_ to $C_{N+1}$ [11]
λ	1.0 × 10^5^	*s* ^−1^	Decay rate of phosphorylated free TCRs [27]
ρ	1.0 × 10^3^	*s* ^−1^	TCR-pMHC rebinding rate [27]
*r*	1.5 × 10^0^		Factor in dephosphorylation rate [60]
*r*_*p*_	1.03 × 10^0^		Factor in phosphorylation rate [60]
*Y*_*T*_	1.0 × 10^2^	*molecules/cell*	Total number of Y molecules per T cell [12]
*Z*_*T*_	2.5 × 10^0^	*molecules/cell*	Total number of Z molecules per T cell [12]
σ	1.0 × 10^2^	*s*^−1^ (*molecules/cell*)^−1^	*C*_*N*_-dependent *Y* activation rate [12]
δ	1.0 × 10^2^	*s*^−1^ (*molecules/cell*)^−1^	*Y*-dependent *Z* activation rate [12]
μ	5.0 × 10^2^	*s*^−1^ (*molecules/cell*)^−1^	*C*_*N*_-dependent *Z* deactivation rate [12]
$\overset{y}{\underset{+}{\gamma }}$	1.0 × 10^0^	*s* ^−1^	Basal *Y* activation rate [12]
$\overset{y}{\underset{-}{\gamma }}$	5.0 × 10^2^	*s* ^−1^	Basal *Y* deactivation rate [12]
$\overset{z}{\underset{+}{\gamma }}$	1.0 × 10^0^	*s* ^−1^	Basal *Z* activation rate [12]
$\overset{z}{\underset{-}{\gamma }}$	5.0 × 10^2^	*s* ^−1^	Basal *Z* deactivation rate [12]
*k*_+_	1.0 × 10^0^	*s*^−1^ (*molecules/cell*)^−1^	Association rate constant [28]
*k*_−_	1.0 × 10^1^	*s* ^−1^	Dissociation rate constant [28]

Positive sensitivity values indicate a direct relationship between a state variable and a parameter, while negative values indicate an inverse relationship. However, for the purpose of this work what is relevant is how intense is the sensitivity. Therefore, we calculated in all cases the absolute value of relative sensitivities.

#### (a) Sensitivity of *R*(*t*) to the unbinding rate$k_{\;off}$.

**Fig 2:**
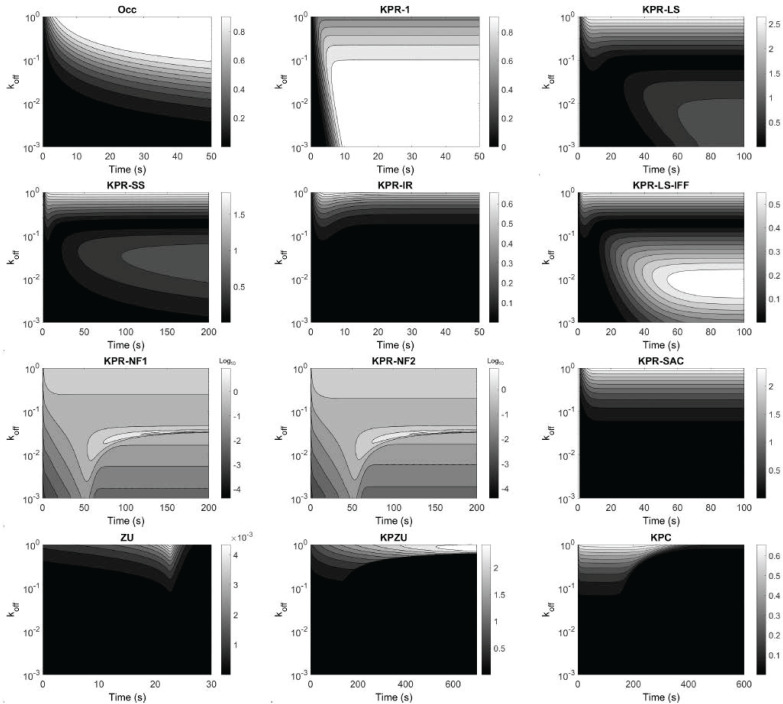
Contour map of the sensitivity of the response *R*(*t*) to variations in the unbinding rate $k_{\;off}$ in each model. Note that the sensitivity gradient bars at the right of each panel are in arithmetic scale, except in the panels corresponding to the *KPR-NF1* and *KPR-NF2* models, where they are in base-10 logarithmic scale. The *ST* model is not included because $k_{\;off}$ is not a parameter in this model.

We calculated how much the dissociation rate $k_{\;off}$ impacts the intensity of the response, *R*(*t*), along a time interval and for a wide range of $k_{\;off}$ values. The results for each model are shown in Fig 2 as panels of ‘hea*t*map’ sensitivity over two variables: time on the horizontal axis and a kinetic parameter on the vertical axis (sometimes plotted on a logarithmic scale). At each point of this grid, the corresponding value is quantified with a gray color code, indicating how much the output changes when the parameter value is slightly changed. Lighter regions correspond to higher sensitivity (*i.e.*, small parameter changes lead to noticeable changes in the output), whereas darker regions correspond to lower sensitivity. Contour lines connect combinations of time and parameter values that have same sensitivities, helping to identify regimes in which the output is most (or least) affected by parameter uncertainty. The time interval was chosen specifically for each model to better capture the sensitivity variations along the range of $k_{\;off}$ values. This provides a *contour map* that allows to pinpoint the $k_{\;off}$ values and times at which sensitivity is highest. The model where *R*(*t*) is less sensitive to $k_{\;off}$ is *ZU*, with a maximum sensitivity in the order of 10^−3^. Models *KPR-IR* and *KPR-LS-IFF* are next, with a maximum sensitivity ≈0.5. On the other hand, the *KPR-LS*, *KPR-SS*, *KPR-SAC*, and *KPZU* models exhibit the highes*t* sensitivity, with a maximum that is 4–5 times higher than that in the previously mentioned models. Nevertheless, those sensitivities are still moderate. Also, we notice that in the negative feedback models (*KPR-NF1* and *KPR-NF2*) the highest values of the sensitivity of *R*(*t*) to $k_{\;off}$ are restricted to a very small range of values of that parameter.

In general, the sensitivity of *R*(*t*) to $k_{\;off}$ strongly depends on the specific values of $k_{\;off}$ and less on the specific time, except in the *KPR-LS-IFF* and *KPZU* models, where it also depends strongly on the specific time. Since $k_{\;off}$ quantifies the average ligand binding lifetime (or dwell time), sensitivity to $k_{\;off}$ quantifies how much *R*(*t*) changes when ligand dwell time changes just a little, and this is analyzed within a large range of possible dwell time values and at different times after the start of the TCR-pMHC in*t*eractions. In the basic *KPR-1*, this effect is time-invariant after an initial transient, biologically meaning that ligand lifetime controls *R*(*t*) in a similar way over time, once signalling has accumulated. In contrast, models that include regulatory motifs show time-dependent as well as dwell time-dependent sensitivity; accordingly, the impac*t* of dwell time differs between early and late reaction times stages and between low, middle and high ligand dwell times.

#### (b) Sensitivity of *R*(*t*) to the binding rate $k_{\;on}$.

Next, we analyzed the sensitivity of the response to the TCR-pMHC binding rate. Interestingly, all models follow a similar trend: the sensitivity is low (*KPZU, KPC* and *ST* models) to very low (the rest of the models). The results are summarized in Fig D in S1 Text. In the models with very low sensitivity, the maximum values ranged from 0.07 to 0.53 and were attained only during the first few seconds of simulation and for very low $k_{\;on}$ values.

The sensitivity results for the *KPZU* and *KPC* models differ significantly from the other models, with the following three features standing out: (i) within the lower values of the analyzed $k_{\;on}$ range the response is moderately sensitive to changes in $k_{\;on}$ for most or all of the simulation time; (ii) the sensitivity slightly increases over time; and (iii) at high parameter values, changes in $k_{\;on}$ has minimal impact on the response at all simulated times. Finally, in the *ST* model, the sensitivity of *R*(*t*) to $k_{\;eff}$ is qualitatively similar to most models, but for low values of $k_{\;eff}$ the sensitivity is highest at all plo*t*ted times, and for the first ten seconds the sensitivity is highest for the whole plotted range of $k_{\;eff}$.

#### (c) Sensitivity of *R*(*t*) to the phosphorylation rate *k*_*p*_.

**Fig 3:**
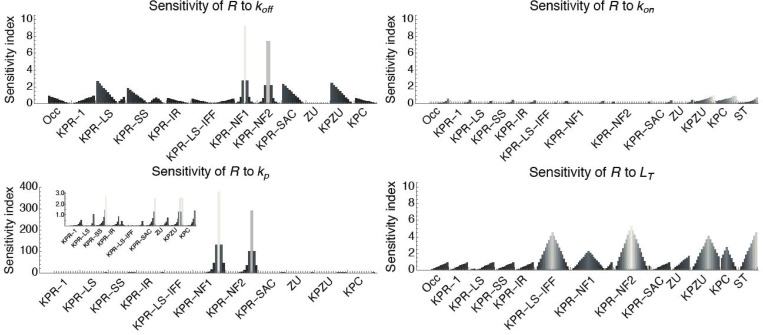
Summary of the sensitivity of the response *R*(*t*) to the three main kinetic parameters, $k_{\;off}$,$k_{\;on}$ and *k*_*p*_, and to the ligand density per cell, *L*_*T*_, in the different models. Data was taken from the graphs in Fig 2, and Fig D to Fig F in S1 Text. For each graph, a corresponding histogram was obtained using the sensitivity values at a particular time at which maximum sensitivity is attained. In each histogram the bars from left to right correspond to the sensitivity values from the maximum parameter value (top of the graph) to the minimum parameter value (bottom of the graph) at that particular time. Note the very high sensitivity of *R*(*t*) to *k*_*p*_ in models KPR-NF1 and KPR-NF2 compared to the other parameters. In that graph an inset is included with a scale appropriate for visualizing the sensitivity in the other models.

The sensitivity of the response to the phosphorylation forward rate, *k*_*p*_, follows a pattern qualitatively similar to that of the sensitivity to $k_{\;on}$, but with the highest sensitivity attained at all times and, as a general rule, for higher values of *k*_*p*_ compared to $k_{\;on}$. The results are shown in Fig E in S1 Text and summarized in Fig 3. Notably, for the *KPR-NF1* and *NF2* models a very short range is observed, within which the response becomes highly sensitive to variations in *k*_*p*_; in other words, there is a critical value of *k*_*p*_ (*k*_*p*_ ≈ 1.2 s^−1^) that not only maximizes its impact on the response, but that impact is 50- to 60-fold higher than the highest sensitivity to any of the other parameters. Below this critical value, the sensitivity to *k*_*p*_ becomes moderate at all times. Another interesting case is the *KPR-LS-IFF* model, whose response is maximally sensitive to *k*_*p*_ within two distinct ranges of this parameter, separated by a wide and deep basin. Finally, the case of the *KPZU* model is qualitatively like that of the *KPR-NF1* and *NF2* models, but with a wider central range, where the highest sensitivity, observed at *k*_*p*_ ≈ 1.25, is moderate.

#### (d) Sensitivity of *R*(*t*) to total ligand *L*_*T*_.

With respect to the sensitivity of the response to the total ligand or antigen dose, *L*_*T*_, the models *Occ*, *KPR-1*, *KPR-LS*, *KPR-SS*, *KPR-IR*, *KPR-SAC*, and *ST* follow a quite similar pattern and have a maximum sensitivity ≈1, except in the *ST* model, where it is ≈4. In all these models, except *KPR-SS*, at any given ligand density higher than 10^3^ molecules/cell, the sensitivity decreases with time, initially fast and then gradually slower. At low ligand densities, the sensitivity is not only highest but independent of time during all the computed time interval (Fig F in S1 Text). In the *KPR-SS* model, the sensitivity is nearly constant in time at any given ligand density. In these seven models, at any time after the first two seconds, the sensitivity increases steadily from near zero to a maximum value for decreasing ligand density (Fig F in S1 Text). The *KPR-LS-IFF* model differs substantially from the above models in that the sensitivity of *R*(*t*) to *L*_*T*_ is biphasic at any given time after the first few seconds, exhibiting a maximum at intermediate ligand densities (200–500 molecules/cell) *t*hat decreases with time. The *KPC* model displays a similar biphasic behavior, but here the sensitivities are largely independent of time, and the maximum sensitivity is attained at *L*_*T*_ ≈ 100 molecules/cell. The *KPR-NF1* and *NF2* models also have a biphasic behavior of the sensitivity of *R*(*t*) to *L*_*T*_, but only within a time window from about 20 *s* to 70 *s* and a very small range of ligand density, located near the highest values of *L*_*T*_. Finally, the *ZU* and *KPZU* models display a very low sensitivity for *L*_*T*_ < 10 molecules/cell at all computed times. On the other hand, in the *ZU* model, *t*he sensitivity increases for decreasing values of *L*_*T*_ and for increasing time. In contrast, the *KPZU* model displays a biphasic behavior with a maximum sensitivity at *L*_*T*_ ≈ 5 molecules/cell that increases with time.

The sensitivities of *R*(*t*) to parameters $k_{\;off}$, $k_{\;on}$, *k*_*p*_ and *L*_*T*_ are summarized as histograms in Fig 3. For each model, histograms were obtained from data at a time when maximum sensitivity is attained for some value of the concerned parameter. At other times, the sensitivities were either similar but lower or much lower.

#### (e) Sensitivity of *R*(*t*) to *b* (spontaneous dephosphorylation rate constant for *C*_*i*_ complexes), γ (rate constant for SHP-1-dependent dephosphorylation of *C*_*i*_ complexes) and *P*_*T*_ (total SHP-1 molecules).

The *KPR-NF1* and *KPR-NF2* models introduce a passive or spontaneous dephosphorylation of *C*_*i*_ complexes to a *C*_*i*−1_ state, and an active dephosphorylation mediated by active *SHP-1* phosphatase. Disclosing the impact that the parameters involved in such dephosphorylation process have on the response could be valuable for explaining how SHP-1 cell level impacts the efficiency of antigen discrimination, which is an open question [25]. To shed light on this aspect, we performed additional sensitivity analyses for these two models. Two parameters, *b* and γ, and one state variable, *P*(*t*) (active *SHP-1*), determine the *C*_*i*_ dephosphorylation rate; in addition, for fixed rates of the dynamics of *SHP-1* in those models, the level of *P*(*t*) is de*t*ermined by *P*_*T*_, the total cellular amount of *SHP-1*. Hence, we analyzed the sensitivity of *R*(*t*) to parameters *b*, γ and *P*_*T*_. Results are shown in Fig G(a) in S1 Text, left and middle panels.

In spite of the different definitions of *R*(*t*) in *KPR-NF1 KPR-NF2*, the sensitivities are very similar in both models. Specifically, *R*(*t*) has a quite modest sensitivity to γ, with maximum values of 2.72 for *KPR-NF1* and 2.14 for *KPR-NF2* (Fig G(b) in S1 Text). In contrast, the maximum sensitivities to *b* and *P*_*T*_ are, respectively, approximately 10- and 300-fold higher than that to γ (Fig G(b) in S1 Text). However, such maximum sensitivities to *b* and *P*_*T*_ are attained only within a very narrow range of parameters’ values (Fig G(a) in S1 Text), close to their reference values (Table 3).

#### (f) Sensitivity of *P*(*t*) (active SHP-1 level) to$\;k_{\;off}$, *b* and γ.

Given the high impact of *k*_*p*_ on the response (Fig 3), the direct effect of *C*_1_ levels on the generation of active *SHP-1*, and the direct effect of *b*, γ and *P*(*t*) on the generation of *C*_1_ from *C*_2_ (Fig A(g) in S1 Text), we hypothesized that *b* and γ could also have an importan*t* impact on the levels of *P*(*t*). Since $k_{\;off}$ has also a direct effect on the levels of *C*_1_, we considered that it could also have an important impact on the levels of *P*(*t*). Hence, we inves*t*igated the sensitivity of *P*(*t*) to $k_{\;off}$, *b* and γ. Because the definition of *P*(*t*) is the same in both models, the results apply to bo*t*h of them. For these three parameters the sensi*t*ivity has a triphasic behavior after the first 50 seconds (Fig G(a) in S1 Text). Thus, for a fixed time, and going from highest to lowest parameter values, *t*he sensitivity first decreases, then increases until a maximum, and finally decreases again. The maximum sensitivity to $k_{\;off}$ and γ are moderately high, respectively, 6.48 and 5.05 (Fig G(b) in S1 Text right panel). However, the maximum sensitivity of *P*(*t*) to *b* is quite high, about 6-fold higher than that to $k_{\;off}$ and γ.

## Discussion

Identifiability analysis is being increasingly used in theoretical biology, particularly in biochemical networks and systems biology, to assess whether a mathematical model is well formulated to provide reliable predictions [15–19,40]. However, in the immunological field modeling works have rarely analyzed parameter identifiability (*e.g.*, [41–43]) and models of basic immune processes, such as lymphocyte activation or the germinal center reaction, to the best of our knowledge have not yet been analyzed in this respect. In contrast, sensitivity analysis is being increasingly performed also in the study of models of basic immune processes (*e.g.*, [20,44]). In the present study, we have analyzed thirteen *phenotypic* models of T-cell activation from the point of view of parameter identifiability and state variables’ sensitivity to parameters.

### Identifiability of T-cell activation models

Identifiability analysis assumes a given known or measured output. We considered here the following *known output* configurations: (1) five individual output variables (*T*(*t*), *T*_*T*_, *R*(*t*), *E*_*max*_ and *EC*_50_), (2) a combination of two individual outpu*t* variables (*e.g.*, *R*(*t*)+*T*(*t*)), and (3) a combination of one or two output variables with parameters $k_{\;on}$ and/or *k*_*p*_ (*e.g.*, $R(t)+T(t)+k_{\;on}+k_{p}$). Our resul*t*s indica*t*e that the best output configurations, in the sense that render identifiable the largest number of the analyzed models, are *R*(*t*)+*T*(*t*) and *R*(*t*) + *T*_*T*_, while the worst configurations, in that sense, are *T*_*T*_, *E*_*max*_ and *EC*_50_ (summarized in Table D in S1 Text). Moreover, our results make clear that, in general, to improve the model’s identifiability, $k_{\;off}$ is the most important parameter *t*o be de*t*ermined, in addition to the outputs *R*(*t*) and *T*(*t*). In contrast, knowing $k_{\;on}$ and/or *k*_*p*_ in addition to any of the considered outputs does not improve the model’s identifiability.

Lever *et al.* systematically derived and analyzed many models of T-cell activation. Their results suggested that the *KPR-LS-IFF* model is the one that best captures all phenotypic traits analyzed by them [12]. However, our identifiability analysis indicates that the model is non-identifiable under any feasible output configuration. More specifically, parameter δ and the values *Y*(0) and *Y*_*T*_ of the trigger of T-cell activation remain non-identifiable under all output configurations. This, together with the fact that *Y* is a hypothetical metabolite, hampers the reliability of this model for predictive purposes. A similar case is that of the *KPR-NF1* and *KPR-NF2* models. The *KPR-NF1* model was proposed by Altan-Bonnet *et al.* to account for the observed important impact of *SHP-1* on T-cell activation [25,35]. Several years later a further modification was proposed, named here the *KPR-NF2* model, to account for recent observations indicating that the number of available ITAMs in TCRs has an important impact on the kinetics of the T-cell response [2]. However, similarly to the *KPR-LS-IFF* model, our present analysis indicates that the parameter γ and the values of *P*(0) and *P*_*T*_ that define the negative feedback modulus are non-identifiable under any output configuration examined (S1 Text, Table A and Table B), thus affecting the reliability of these models in practice. Nevertheless, in this case this could be remedied by measuring additionally the parameter γ as well as *P*_*T*_ and *P*(0) (the amount of active *SHP-1* in resting conditions).

Besides the merits of the different models in coping with the three key features of sensitivity, specificity, and reaction speed, the question of which of the proposed models is better to reliably predict the response of T cells to antigen must be answered considering identifiability and observability issues. For instance, if both the number of individual outputs and the total number of output configurations that make a model identifiable are taken as a measure of how robustly a model is defined, our results indicate that the *KPR-IR* model is better than others, because it is identifiable not only under all combined output configurations but also under all individual outputs (Table 2 and Table C in S1 Text). The *KPR-IR* model was originally proposed [27] to enhance the poor sensitivity of the *KPR-1* model while retaining specificity; to this end it requires a high number of proofreading steps (*N* ≥ 21), otherwise the kinetics of the response would be very similar to that of the *KPR-1* model [27,23]. Here we analyzed a simpler *KPR-IR* version [24] for which, unlike the *KPR-1* model, all parameters are at least SLI under all output configurations, and SGI when *R*(*t*) (alone or together with *T*(*t*) or *T*_*T*_) or *T*(*t*) together wi*t*h *k*_*p*_ are known, supporting the relevance of this model. The next bes*t* models are *KPR-SS* and *KPC* with seven output configurations that make them identifiable, followed by the *KPR-SAC*, *ZU*, and *KPZU* models (summarized in Table C in S1 Text).

### Sensitivity in T-cell activation models

Similarly to identifiability, sensitivity to parameters was not analyzed in any of the original articles of the models studied here. However, the combination of sensitivity and identifiability analyses is a powerful tool to determine model parameters that could be used to control or fine tune a T cell response. There are four potential scenarios that stand out: (1) the response *R*(*t*) has moderate sensitivity to a parameter that is identifiable; then, that parameter is a good candidate for controlling the response; (2) the sensitivity of *R*(*t*) to a parameter is low or very low within a range of biologically relevant values; then, even if the parameter is identifiable, its specific value will affect very little the predicted output and hence that parameter cannot be used to control it; (3) the sensitivity of *R*(*t*) to a parameter is high or very high; in this case even small changes in its value will greatly impact the response, making that parameter useless to control it; and (4) a model has some non-identifiable parameters under all output configurations, but the sensitivity of the response to those parameters is very low; then, that model can still be used to make reliable predictions.

The sensitivity analysis to parameters of many of the models analyzed in the present work has been performed, for the first time to our knowledge, besides our own work, in a very recent paper [22]. However, the method used in that paper (Latin Hypercube Sampling-Partial Rank Correlation Coefficient (LHS-PRCC)) is significantly different from the method we use and answer different questions. Thus, the LHS-PRCC method varies several parameters simultaneously, and ranks those that most consistently increase/decrease the output across the explored parameter space. In contrast, our one-at-a-time complex-step approach quantifies the effect of perturbing a single parameter while keeping the remaining parameters fixed at nominal values that represent a given experimental setting. For several of the analyzed models shared by that work and ours a drawback of applying LHS-PRCC is that this method detects correctly only monotonic relationships. If the output follows a bell-shaped or bimodal behavior, like in several of the analyzed models, it can yield low sensitivity values for a given parameter even when the parameter has in fact a large impact on the response [45].

Our sensitivity analyses allowed us to identify in each model which of the four main parameters, that is, the unbinding rate, $k_{\;off}$, the binding rate, $k_{\;on}$, the phosphorylation rate, *k*_*p*_ and total ligand, *L*_*T*_, have a moderate impact on the response over time (*R*(*t*)), and the range at which they attain that sensitivity, such that variations in their values can lead to comparable changes in the response. If we focus on *t*he three main kinetic parameters that are common to most T-cell activation models (i.e., $k_{\;off}$, $k_{\;on}$ and *k*_*p*_), our results clearly show that $k_{\;off}$ has quite moderate impact on the response in most models. With respect to the phosphorylation rate *k*_*p*_, with the exception of the *KPR-NF1* and *KPR-NF2* models (where the maximum sensitivity of *R*(*t*) to *k*_*p*_ is very high, but only within a very narrow range of *k*_*p*_ values), this parameter also has a moderate effect on the response (*R*(*t*)) in the other eleven models and at almost any time step. We investigated further what could be special in the *KPR-NF1* and *KPR-NF2* models such that in them *R*(*t*) has so high sensitivity to *k*_*p*_. We found that of the parameters involved in the negative feedback modulus only the level of to*t*al *SHP-1* (*P*_*T*_) has also a very high impact on *R*(*t*). Thus, all the three main parameters affecting the level of the complex *C*_1_ (the activator of *SHP-1*) have the greatest impact on *R*(*t*). As for *t*he sensitivity to $k_{\;on}$, there is a remarkable difference between the moderate maximum sensitivity in the *KPZU*, *KPC* and *ST* models and the low maximum sensitivities in the o*t*her models. In other words, $k_{\;on}$ has in general a very limited impact on *R*(*t*). Interestingly, in the *KPZU* and *KPC* models the sensitivity of the response to $k_{\;off}$ and $k_{\;on}$ is somehow opposite to each other, thus, while it is very low to $k_{\;off}$ within a large parameter range, it is modera*t*e to $k_{\;on}$ also within a large parameter range. Finally, we have shown here that the sensitivity of *R*(*t*) to the total ligand per APC, *L*_*T*_, is low *t*o moderate in all models but stronger than to $k_{\;off}$ in all models (Fig 3). Interestingly, comparing the *Occ* and *KPR-1* models shows that proofreading *per se* does not change the range of sensitivity values of *R*(*t*) to *L*_*T*_, but mainly strengthens the contribution of $k_{\;off}$; in contrast, large ranges of the sensitivity of *R*(*t*) to *L*_*T*_ are associated with additional regulatory motifs (*e.g.*, those in KPR-LS-IFF, KPR-NF, ZU, KPZU, and KPC models). We remark that our sensitivity analysis is explicitly restricted to early T-cell activation dynamics on a seconds-to-minutes timescale, as captured by the phenotypic models analyzed here [11,13]. Hence, processes operating on longer timescales, like T-cell differentiation, are outside the modeling framework.

In the above mentioned paper, [22], it was found that parameter *k*_*p*_ has a strong impact on the response, *R*(*t*), in all analyzed models. This is in contrast to our finding *t*hat *k*_*p*_ has a moderate impact on the response in all models, except the *KPR-NF* ones, where it has a very high impact (see above). However, in agreement with out findings, that paper reports that *L*_*T*_ (ligand concentration) has also a significant impact on the response. Nevertheless, it must be noted that a direct comparison between the results in [22] and ours should be made with great caution because of the different methods used for the sensitivity analysis.

The response level depends essentially on the more or less intricated way the different biological processes implemented in each model are interrelated, which makes very difficult if not impossible to unravel unambiguously why some parameters have on the whole a larger impact on the response than others. Nevertheless, some general remarks can be made concerning the common core kinetic parameters, $k_{\;on}$, $k_{\;off}$ and *k*_*p*_. First, in the design of most models $k_{\;on}$ plays a minor role in the generation and accumulation of *C*_*N*_ complexes, which define or determine the response. In contrast, the accumulating amount of *C*_*N*_ complexes is increasingly determined with the number of proofreading steps by the interplay of unbinding and proofreading rate constants, respectively, $k_{\;off}$ and *k*_*p*_, in a non-trivial way —even in the simplest KPR model, the *KPR-1* one. This is in close agreement with a previous analysis of effective $k_{\;on}$ ($\overset{\;eff}{\underset{\;on}{k}}$) and $k_{\;off}$ ($\overset{\;eff}{\underset{\;off}{k}}$) rate constants experimentally estimated in conditions close to *in vivo* 2 dimensional (2D) TCR-pMHC interactions, which indicate clearly that the sensitivity of the T cell response to ligands was substantially higher to 2D $\overset{\;eff}{\underset{\;off}{k}}$ than to 2D $\overset{\;eff}{\underset{\;on}{k}}$ [34,46]. Second, our results also show that in general, except in the *KPR-NF* models, the sensitivity of the response to *k*_*p*_ is quite similar to the sensitivity to $k_{\;off}$. This indicates that, despite the additional regulatory motifs and the new rates incorporated with them, the impact of $k_{\;off}$ and *k*_*p*_ on the time course of the response is little affected, and hence is quite robust. Last, the sensitivity results agree with and corroborate the hypotheses on which some models were built. For example, the *zero-order ultrasensitivity* mechanism is designed to improve the receptor’s sensitivity to changes in ligand concentration, in the sense of generating a steeper dose-response curve: there is an operating window around a low threshold in which small variations in *L*_*T*_ produce large changes in response. Consistently, in the heatmap of the models incorporating this mechanism, high sensitivity to *L*_*T*_ is concentrated near the threshold and decreases outside it because, at low doses, the response is nearly zero, and at high doses, the system enters a saturation regime in which *R*(*t*) changes little in response to further increases in ligand. This happens more conspicuously for the *KPZU* and *KPC* models.

In summary, in most models the response may be best regulated by tuning $k_{\;off}$, *k*_*p*_ and *L*_*T*_ provided they remain within a range not too far from the maximum sensitivity of *R*(*t*) to them (Fig E in S1 Text). Interestingly, that range is large in nearly all models. In contrast, in general, except the *KPZU*, *KPC* and *KPR-ST* ones, $k_{\;on}$ is no*t* a good candidate to control the response because the sensitivity of *R*(*t*) to it is very low. Finally, in the case of the *KPR-IR* model —the one favored by identifiability analysis— the maximum sensitivities of *R*(*t*) to $k_{\;off}$, $k_{\;on}$, *k*_*p*_, and *L*_*T*_ are, respectively, 0.66, 0.31, 0.86, and 0.91. This suggests tha*t* in this model, the parameters *k*_*p*_ and *L*_*T*_ are particularly well suited to tune the response.

## Conclusion

The older phenotypic models (*Occ* and *KPR-1*) could only account for a limited subset of observed T-cell phenotypes. Variants of those models were then developed to incorporate an increasing number of those phenotypes. As shown here, some of them are identifiable when *R*(*t*) or *T*(*t*) are the only known output. Moreover, within the output configurations and parameter values considered in this study, the *KPR-IR* was revealed as the most suitable model for predicting and potentially controlling T-cell responses. The reason is that it remains structurally identifiable as a whole across all considered measurement scenarios, while preserving considerable sensitivity to all parameters, particularly the ligand dose, *L*_*T*_, and the proofreading rate, *k*_*p*_, which influences the time to activation. We remark, however, that this conclusion should be interpreted under the scope and configurations adopted in our analysis.

Further modifications of identifiable, KPR-based models, devised to more comprehensibly capture the diverse T-cell phenotypes, would likely exhibit identifiability issues. However, such issues should not lead to their automatic rejection: as we have found, some of the non-identifiable models can become identifiable by measuring a second output; moreover, as indicated above for the *KPR-NF1* and *KPR-NF2* models, structural identifiability issues of T-cell activation models can be remedied in some cases by measuring still more outputs. When this solution is not experimentally feasible or convenient, an alternative may be to simplify or reparametrize the equations of the model [17]. Lastly, we note that the findings reported here have been obtained with a structural identifiability approach that implicitly assumes the availability of noiseless data, collected at as many time points as necessary. This is of course an ideal setting; real experimental setups introduce additional limitations, which can be accounted for with practical identifiability techniques [47].

Any immune system deficiency in discriminating between tumor- or pathogen-derived Ags and normal self-protein Ags can lead to pathologies such as cancer or persistent viral infections (tolerance or low response level) or autoimmunity (excess reactivity). Having a model of T lymphocyte activation with reliable predictive capacity in different experimental situations would be instrumental in the development of theoretically based methods to control the immune response and make it a more efficient process in the face of pathological threats. Incorporating identifiability and sensitivity analysis in the study of models of fundamental immune processes should greatly enhance the values of those models and the immunological insights that can be gained from them.

## Materials and methods

### Modeling framework and notation

We consider here deterministic models described by ordinary differential equations (ODE) of the form:


$$ℳ:\left\{ \begin{array}{c} \begin{array}{ccc} x(t) & = & f(x(t),\theta ,u(t)),\\ y(t) & = & h(x(t),\theta ,u(t)),\\ x(0) & = & x^{0} \end{array} \end{array}\right.$$ (4)

where $x(t)=(x_{1}(t),\ldots ,x_{n}(t))$ is the state variables vector, $u(t)=(u_{1}(t),\ldots ,u_{q}(t))$ is the input variables vector (usually, in biological models there are no input variables), $\theta =(\theta_{1},\ldots ,\theta_{p})$ is the parameters vector, $x(0)=(x_{1}(0),\ldots ,x_{n}(0))$ is the initial conditions vector, and $y(t)=(y_{1}(t),\ldots ,y_{m}(t))$ is the output vector. Since this last vector corresponds to experimentally measurable functions, it is usually, but not always, a subset of the state variables. All the components of the four vectors take real values. The functions f(x(t),θ,u(t)) and h(x(t),θ,u(t)) considered in this work are generally non-linear, and contain rational terms. The output is known, while the parameters (or a subset of them) are typically unknown. To simplify the notation we sometimes omit the dependence on *t*.

### Structural identifiability and observability analysis

In order to ascertain whether model parameters can be uniquely determined from the outputs, i.e., from experimentally measured quantities, we perform a *Structural identifiability* analysis (SIA) [39]. A parameter with an infinite number of values compatible with the experimental data is structurally unidentifiable, as its true numerical value cannot be determined. Conversely, a parameter is globally identifiable if there is only one possible solution in the parameter space, and locally identifiable if there are a finite number of solutions.

*Observability* is a property that informs about the possibility of inferring the internal state of a system at time *t* by observing its output at times τ such that t≤τ. Notice that, by considering the unmeasured initial conditions *x*^0^ as parameters, their observability can be studied as structural identifiability, *i.e.*, identifiability of an initial condition *x*^0^ is equivalent to observability of *x*_*i*_(*t*) [19]. Thus, we assessed the observability of state variables *x*(*t*) by analysing the identifiability of their initial conditions *x*^0^.

Structural identifiability was assessed using a number of methods. Whenever possible, we followed a differential algebra approach that can be applied to systems defined by rational ODE functions, and involves deriving algebraic equations that connect the model parameters with the inputs and outputs [48]. Briefly, model equations (4) are transformed into a set of polynomial differential equations that depend solely on the output variables *y* [49], preserving the dynamics of the model output while removing the state variables. Extracting from these equations the coefficients that multiply the same variables in terms of *y* and grouping them into the same equation, we obtain the so-called exhaustive summary. Then, the identifiability of model parameters can be determined by evaluating whether the mapping defined by the exhaustive summary is unique [50]. The differential algebra approach can distinguish between global and local identifiability. To apply it we used SIAN (Structural Identifiability ANalyser), an open-source tool that integrates differential algebra with a Taylor series-based method [51,52]. This choice was motivated by SIAN’s computational efficiency, reliability, and its ability to include the initial conditions of state variables, *x*^0^, as parameters in the analysis [53]. In those cases where the analysis could not be performed with SIAN due to non-rationalities, we used alternative methods that are more generally applicable, but can only determine local identifiability: STRIKE-GOLDD [54] and StrucID [55].

### Output variables

Structural identifiability and observability depend on the output definition. Hence, in our analyses we considered different possible output configurations for each model, including, as mentioned above, the total amount of TCRs per cell (*T*_*T*_), the total amount of free TCRs per cell (the state variable *T*), the total amount of triggered or activated TCRs per cell (which is *R*(*t*) except in one model, see below), the maximum achievable T-cell activa*t*ion level or maximum *R*(*t*) (defined in most of the considered models as *E*_*max*_ = *R*(*t*) *at steady state and saturating conditions of pMHC*), and the amount of pMHC per APC that results in 50% of *E*_*max*_ (*EC*_50_).

In each model, the first two equations of the ODE system govern the dynamics of free receptors and ligands. Therefore, the outputs *T* and *T*_*T*_ are common to all models. However, the definition of *R*(*t*) is not the same in all models. Thus, while in most pheno*t*ypic models *R*(*t*)= *amount of TCRs in ligand-receptor complexes that reach the signaling state* per T cell, in some models (*KPR-SS* and *KPR-IR*) *R*(*t*) also includes the amount of recently unbound TCRs *t*hat remain in the signaling state. This complicates considerably the calculation of *E*_*max*_ and *EC*_50_ in several of the models. In Box 1 we outline the main steps we followed to derive the equations for *E*_*max*_ and *EC*_50_ in most of the models. A detailed description of the derivation of *E*_*max*_ and *EC*_50_ for each phenotypic model is provided in [24].

**Box 1. Outline of the procedure for calculating *E*_*max*_ and *EC*_50_ in T-cell activation ODE models.**

*Step 1: Conservation equations for ligands and receptors:* We assume that the total amount of pMHC and TCR does not change during the T-cell activation process. Therefore, denoting *L*_*T*_ = total cognate pMHC; *L* = total unbound cognate pMHC; *T*_*T*_ = total TCR; *T* = total unbound TCR; and *C*_*T*_ = total TCR-pMHC complex in the system, the following conservation equations hold:

$$\begin{array}{cc} L_{T} & =L(0)=L(t)+C_{T}(t)\\ {}[5pt]T_{T} & =T(0)=T(t)+C_{T}(t)\\ \left[\mathrm{-2pt}\right] \end{array}$$ (5)

*Step 2: Calculate* $\hat{R}$*, the response level R(t) at steady state, as a function of* *L*_*T*_, *T*_*T*_ *and* θ.

*Step 3: Calculate* *E*_*max*_ *as* $\lim_{L_{T}\to \infty }\hat{R}$*.* In mathematical terms, ligand saturation is expressed as *L*_*T*_ → ∞. Under these conditions, most model systems reach their maximal efficacy, allowing the response to be evaluated at its upper limit.

*Step 4: Calculate EC*_*50*_*.* The ligand potency or *EC*_50_ is defined as the value of the total amount of ligands, *L*_*T*_, at which $\hat{R}=E_{max}/2$. Replacing $\hat{R}$ and *E*_*max*_ with their expressions (obtained in steps 2 and 3) yields, in most cases, an expression for *L*_*T*_ in terms of *T*_*T*_ and θ.

### Sensitivity analysis

We apply sensitivity analysis to quantify the extent to which a model prediction of T-cell activation depends on each parameter. More specifically, we perform a local sensitivity analysis to calculate quantitative changes in model outputs relative to parameter variations around a local point in the parameter space [56]. For that, we define first a large range of values, spanning several orders of magnitude, to assure the true biological value of the parameter under study is included (Table E in S1 Text); and then, we evaluate the sensitivity on a fine grid across $\left[\theta_{j,\min},\theta_{j,\max}\right]$, while keeping all other parameters fixed at their nominal values (Table 3). We do that by first computing at each time point *t*_*k*_ (*k* = 1, 2, ..., *N*) the absolute sensitivity coefficient of output variable *y*_*i*_ to parameter $\theta_{j}$, defined as [57,58]:


$$s_{ij}(t_{k})=\frac{\partial y_{i}(t_{k},\bar{\theta })}{\partial \theta_{j}}=\lim_{\Delta \theta_{j}\to 0}\frac{y_{i}(t_{k},\theta_{j}+\Delta \theta_{j})-y_{i}(t_{k},\theta_{j})}{\Delta \theta_{j}}$$ (6)

where $\bar{\theta }$ is the nominal parameter vector. Then, in order to compare the sensitivities to the different parameters of all output values, we normalize them, that is, we obtain the relative sensitivities [21]:


$$S_{ij}(t_{k})=\frac{s_{ij}(t_{k})\cdot \theta_{j}}{y_{i}(t_{k},\theta_{j})}$$ (7)

Absolute sensitivities were calculated following the method presented in [59], which approximates the gradients from (6) by extending the output function to the complex space (see S1 Text, subsection 1.2). We used our own Matlab implementation of the code described in [59], adjusting the presentation of the results to produce plots that better capture how the impact on the response evolves with time. Since the relevant time interval may differ for each model, we explored a sufficiently broad range to capture the model dynamics before reaching the steady state. The code for these calculations is available on GitHub (https://github.com/Xabo-RB/Inmunology-analysis.git).

Unlike structural identifiability analysis, which can be performed symbolically, sensitivity analysis entails integrating ordinary differential equations, which requires assigning numerical values to the parameters. The values used in our analyses are shown in Table 3. In this table we indicate, for each parameter, the value that we used and the reference from which that value was taken.

## Supporting information

S1 Text Supplementary information. Text file that provides theoretical background of the methods applied in this paper, the equations and diagrams of all models, and supplementary results.
(PDF)
